# Case Report: Adjuvant Radiotherapy Can Be an Effective Treatment for Intimal Sarcoma of the Heart

**DOI:** 10.3389/fonc.2021.621289

**Published:** 2021-02-26

**Authors:** Anna Romanowska, Ewa Lewicka, Grzegorz Sławiński, Hanna Jankowska, Renata Zaucha

**Affiliations:** ^1^ Department of Oncology and Radiotherapy, University Clinical Centre, Gdańsk, Poland; ^2^ Department of Cardiology and Electrotherapy, Medical University of Gdańsk, Gdańsk, Poland; ^3^ Department of Noninvasive Cardiac Diagnostics, Medical University of Gdańsk, Gdańsk, Poland; ^4^ Department of Oncology and Radiotherapy, Medical University of Gdańsk, Gdańsk, Poland

**Keywords:** cardiac intimal sarcomas, radiotherapy, radiation-induced heart disease, cardiac function analysis, heart radiation dose

## Abstract

Intimal sarcoma of the heart is a sporadic disease, which involves symptoms of cardiac insufficiency due to a fast-growing intraluminal mass. Tumor resection is the first-line treatment, although its location precludes excision with wide uninvolved margins. Despite the aggressiveness of this neoplasm and a high risk of recurrence even after removal by microscopically radical surgery, no standard adjuvant therapy has been established. Chemotherapy is used either as an adjuvant treatment or in cases of advanced disease. In contrast, the use of radiotherapy is rare and usually considered in a palliative setting because the risk of radiation-induced heart disease after high-dose radiotherapy to the heart is significant. Herein, we present the cases of two patients, both diagnosed with cardiac intimal sarcoma, who received irradiation after tumor resection. In both cases, radiotherapy was effective, providing long-lasting local disease control. We regularly monitored cardiac function in both patients to assess the impact of radiotherapy on tumor-free heart structures. The excellent local control of the disease with only mild long-term cardiac dysfunction in both patients suggests that radiotherapy can be a useful treatment modality in this indication.

## Introduction

The incidence of primary cardiac tumors ranges from 0.02% to 0.25% as reported in autopsy studies (1). Approximately 25% of them are malignant tumors, 75% of which are sarcomas. Cardiac intimal sarcoma (CIS), a type of undifferentiated sarcoma, is particularly rare (1). CIS is characterized by rapid aggressive intraluminal growth, symptoms of cardiac insufficiency, and the median survival of 1.5 months in untreated cases. Owing to the rarity of the disease, the standard of care has not been established (2). Given the lack of evidence-based treatment recommendations, any combination of complete tumor resection (including bench resection) with neo- or adjuvant chemotherapy (CTH) and, less commonly, radiotherapy (RTH) is the therapeutic strategy for localized primary cardiac sarcoma (3, 4).

The main concern associated with using RTH for heart sarcoma is the risk of radiation-induced heart disease (RIHD). Therefore, heart tolerance is usually the dose-limiting factor (3). Herein, we present the history of two patients with CIS admitted to the hospital because of the rapid development and progression of cardiac insufficiency symptoms. Initial echocardiography (ECHO) revealed cardiac tumor in each case. Combined treatment consisting of surgical excision and postoperative RTH was carefully followed with regular cardiac assessments. Since both patients achieved long-term survival, we were able to evaluate heart function changes in the context of radiation dose distribution. In both patients, RTH provided local control of the sarcoma with mild cardiac function impairment. Thus, we believe that RTH can be effective and tolerable in this indication.

## Case Description

### Patient 1

A 47-year-old man was admitted to the University Clinical Center of the Medical University in Gdansk in December 2017 due to worsening exercise tolerance, leg swelling, dizziness, and fainting during physical activity. In October 2017, he was diagnosed with pulmonary embolism, for which he received oral anticoagulants (dabigatran), with short-term improvement. Otherwise, the patient’s medical history was unremarkable. ECHO on admission revealed a tumor filling the right ventricle. The patient underwent an immediate surgical resection of the tumor followed by the reconstruction of the right ventricle (RV) with a BioIntegral patch (BioIntegral Surgical Inc., Mississauga, ON, Canada) and tricuspid annuloplasty using the De Vega technique.

Histopathological examination of the resected specimen confirmed CIS with microscopically positive surgical margins. Postoperatively, the patient presented with symptoms of cardiac insufficiency what excluded him from postoperative CTH. Instead, postoperative RTH was proposed to decrease the risk of recurrence after R1 resection. Computed tomography (CT) scanning for RTH planning performed just 7 weeks after the surgery revealed a recurrence in the RV wall. Therefore the patient received RTH with curative intent at a dose of 66 Gy in 30 fractions. Subsequent heart imaging with magnetic resonance (MR) and CT showed radiation-induced abnormalities with no evidence of local recurrence. At 32 months after the initial surgery, the patient was disease-free. He was diagnosed with a second malignancy during the follow-up period, which was radically resected and confirmed as renal clear cell carcinoma (stage pT1b according to TNM 8^th^ edition) (5).

### Patient 2

In September 2016, a 57-year-old man underwent surgical removal of a left atrium (LA) mass that resembled myxoma on ECHO, with pericardial patch reinforcement of the LA. The diagnosis was made after several months of dyspnea, decreasing exercise tolerance, and fainting. Histopathological examination led to the diagnosis of CIS, with microscopically positive resection margins. The patient’s medical history was unremarkable, apart from well-controlled hypertension and hypercholesterolemia. His overall clinical status was good; therefore, adjuvant CTH consisting of four cycles of doxorubicin with dacarbazine was administered.

In December 2017, after the diagnosis of a local recurrence 9 months after the last CTH cycle, the patient underwent another surgery, during which tumor resection was incomplete. Due to the high risk of further progression, postoperative RTH at a total dose of 66 Gy in 30 fractions was administered, leading to complete remission of the LA tumor. After a year, the patient was diagnosed with a metastatic lesion in the retroperitoneal space. Radical resection of metastasis (in January 2019) was followed by the second-line CTH (gemcitabine and docetaxel). In July 2019, due to further disease progression, the patient was offered oral pazopanib, which he continued for 47 months, achieving reasonable control of the disease.

### Radiotherapy Details

RTH planning and treatment were conducted according to the institutional protocol of internal use. The treatment plan for Patient 2 with small residual mass was prepared using a 4-dimensional CT (4D-CT) scan. On the contrary, in Patient 1, 4D-CT did not add any benefit; therefore, we chose a conventional CT scan. Gross tumor volume (GTV) was contoured on the non-contrast-enhanced CT series fused with contrast-enhanced scans and preoperative imaging with the help of an experienced cardio-radiologist. The clinical target volume (CTV) encompassed the GTV with a 20-mm margin adjusted for anatomical structures and areas preoperatively involved by the tumor. Planning target volume (PTV) was created by adding an 8-mm isotropic expansion to CTV, and PTV-boost was created by adding 8-10-mm margin to GTV. The volumes of the target areas are summarized in **Table 1**. Organs at risk were contoured according to institutional and international guidelines (6, 7).

**Table 1:** Dose distribution among cardiac sub-volume and coronal artery categories and target volumes for both patients.

		Heart		
		Patient 1		Patient 2
V20		100%		58%
V30		95.7%		47.6%
V60		20%		2.5%
MHD		52.9 Gy		31.7 Gy
		Left atrium		
Dmax		62.4 Gy		67.5 Gy
Dmean		45.3 Gy		57.8 Gy
V30		80%		100%
		Right atrium		
Dmax		62.7 Gy		57.3 Gy
Dmean		50.3 Gy		27.4 Gy
V30		93.5%		34.5%
		Left ventricle		
Dmax		64 Gy		58.4 Gy
Dmean		53 Gy		39.1 Gy
V30		100%		73%
		Right ventricle		
Dmax		63.7 Gy		56.7 Gy
Dmean		59.5 Gy		14.8 Gy
V30		100%		3.5%
		LMCA		
Dmax		62.4 Gy		59.8 Gy
Dmean		60.4 Gy		56.8 Gy
		Cx		
Dmax		61.4 Gy		66.8 Gy
Dmean		60.4 Gy		60.8 Gy
		LADCA		
Dmax		62.4 Gy		59.6 Gy
Dmean		59.5 Gy		22.6 Gy
		RCA		
Dmax		62.5 Gy		37 Gy
Dmean		58.8 Gy		11.4 Gy
		Lung		
V5		80		64
V20		32.5		34
MLD		16.5		16.2
Volume (cm^3^)	GTV	CTV	PTVboost	PTV
Patient 1	151.5	606.9	441.6	1246.9
Patient 2	3	210.9	35.3	466

CTV, clinical target volume; Cx, circumflex artery; Dmax and mean, maximal and mean dose adequately; GTV, gross tumor volume; LADCA, left anterior descending coronary artery; LMCA, left main coronary artery; MHD, mean heart dose; MLD, mean lung dose; PTV, planning target volume; RCA, right coronary artery; Vx, the volume of a structure receiving the dose of xGy.

Treatment was planned using intensity-modulated radiation therapy with simultaneous integrated boost technique at a dose of 54 Gy for PTV and 66 Gy for PTV boost. Each RTH plan was evaluated by two medical physicists and two specialists in radiation oncology. The RTH plan for Patient 2 met institutional dose constraints. Patient 1 was informed about the high risk of RIHD due to heart dose violation; he accepted this therapy as his only treatment option. RTH was delivered using a linear accelerator (TrueBeam^®^ SN1403 accelerator, Varian Medical Systems Inc., Palo Alto, California, United States).

**Figure 1:**
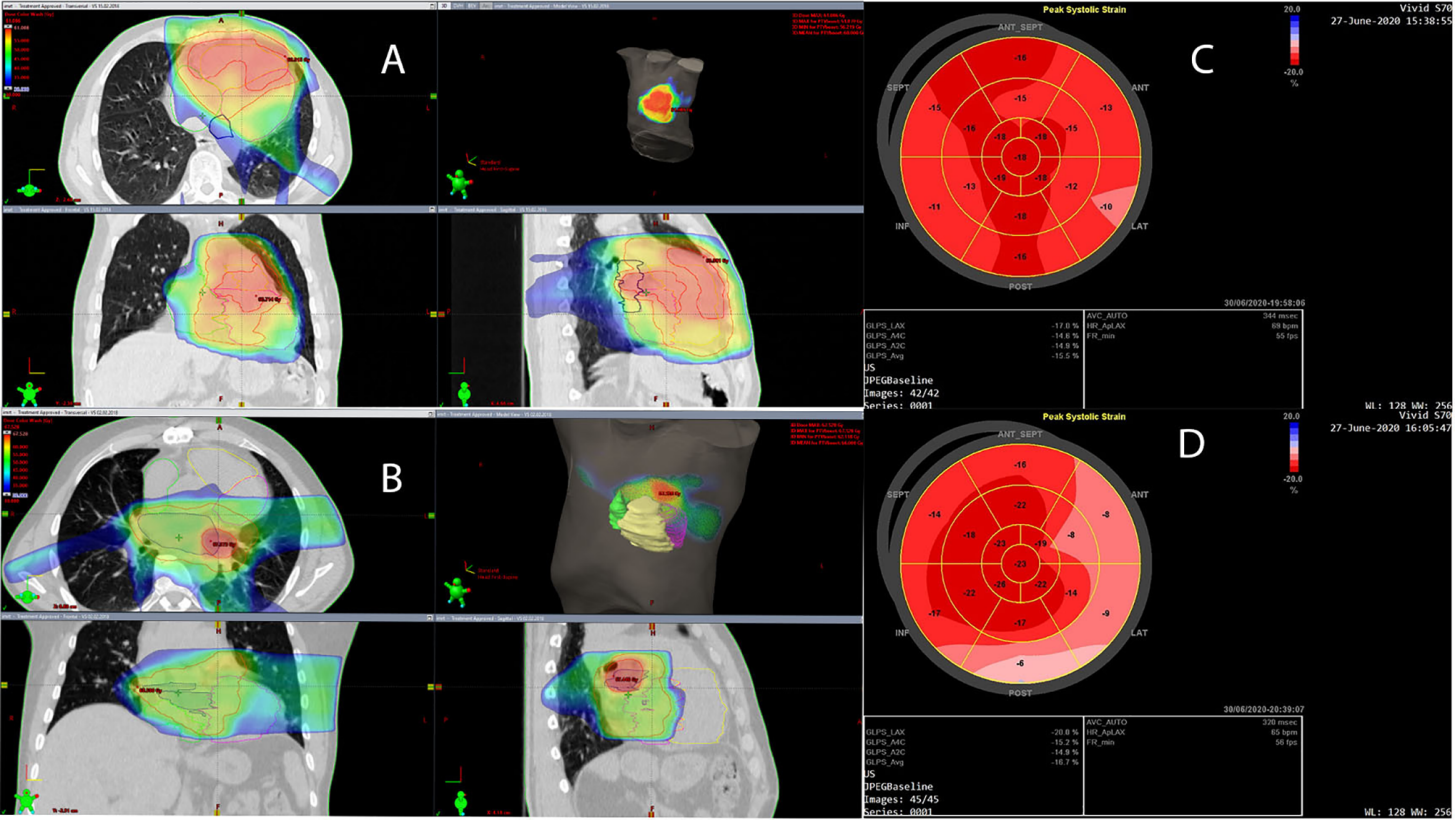
**(A, B)** Dose distribution is presented in dose color wash starting from 30 Gy. Planning target volume (PTV) and PTV boost are indicated with red contours; the left atrium (LA) is indicated with dark blue, the left ventricle (LV) with magenta, the right atrium (RA) with green, and the right ventricle (RV) with yellow. **(C)** Left ventricular global longitudinal strain (GLS) is presented in the bull**’**s-eye diagram obtained with two-dimensional speckle-tracking echocardiography (STE) 26 months after cardiac radiotherapy in a patient with RV intimal sarcoma. The average GLS is -15.5%, and the regional peak longitudinal strain reduction is seen in the basal and partially in the mid-ventricular anterior, lateral, and inferior segments, which may be the result of cardiac irradiation. **(D)** LV GLS in the bull**’**s-eye diagram obtained with two-dimensional STE 27 months after cardiac radiotherapy in a patient with LA intimal sarcoma. The average GLS is -16.7%, however, regional peak longitudinal strain reduction is seen in the basal and partially in the mid-ventricular anterior, lateral, and posterior segments, which may be due to cardiac irradiation.

We retrospectively contoured the heart sub-volumes, including the coronary arteries and heart chambers, following available atlases. Normal tissue radiation doses are summarized in **Table 1**. An example CT scan with dose distribution is presented in **Figures 1A, B**, respectively.

### Cardiac Function

#### Patient 1

The first ECHO examination revealed a large tumor (82 mm × 63 mm) with irregular borders in the RV, filling the RV outflow tract, and penetrating the pulmonary artery with only the peripheral flow preserved. In addition, significant RV enlargement and impaired RV systolic function, right atrial (RA) enlargement, and severe tricuspid valve regurgitation were found (**Table 2**). The serum concentration of brain natriuretic peptide (BNP) was 1165 pg/ml. CT angiography excluded pulmonary embolism. Coronary angiography showed no changes in the coronary arteries. After surgical excision within macroscopically healthy borders of the tumor filling the entire RV, the RV defect was closed using a BioIntegral patch. Concurrently, de Vega annuloplasty for tricuspid regurgitation was performed. The patient received bisoprolol 5 mg daily (QD), torasemide 20 mg QD, eplerenone 25 mg QD, and prophylactic dose of enoxaparin QD. After cardiac RTH (March–April 2018), ECHO, CT, and cardiac magnetic resonance (CMR) imaging showed normal left ventricular (LV) size and function, but RV and RA enlargement persisted. RV systolic function was severely impaired after surgery (**Table 2**), with concentric RV hypertrophy (up to 9 mm). The patient was clinically stable and fully ambulatory. Six months thereafter, RTH pharmacotherapy was modified as follows: ramipril 2.5 mg QD, atorvastatin 40 mg QD, bisoprolol dose was increased to 10 mg QD, rivaroxaban was added, and eplerenone and torasemide were discontinued. Electrocardiography (ECG) showed sinus rhythm and right bundle branch block (RBBB) without other abnormalities. Follow-up ECHO (**Table 3**) (26 months after RTH) showed the enlargement of the RV (5.0 cm), LA (25 cm^2^), RA (25 cm^2^), and mild RV free wall hypertrophy (8 mm). Global LV systolic function was mildly reduced, with LV ejection fraction (LVEF) of 50%, and LV global longitudinal strain (LV GLS) of -15.5% (values <16% considered abnormal). In addition, the hypokinesis of the LV anteroseptal segments was noticed. Analysis of the mitral inflow parameters and pulsed tissue Doppler early diastolic velocities showed preserved LV diastolic function. RV systolic function remained significantly impaired, as indicated by a decrease in the tricuspid annular plane systolic excursion (TAPSE) of 8 mm (norm: >19 mm) and low tricuspid annulus systolic velocity in tissue Doppler (S’RV) of 5 cm/s (norm: >9.5 cm/s). As shown in **Figure 1C**, the bull’s eye diagram of LV GLS demonstrates a significantly reduced peak longitudinal strain in the basal and partially in the mid-ventricular segments. Laboratory tests revealed the normal concentration of high-sensitivity cardiac troponin I and BNP elevated to 942 pg/ml. Thus, the administration of torasemide 20 mg and eplerenone 25 mg was resumed.

**Table 2:** Cardiac dimensions and parameters describing left and right ventricular systolic function in transthoracic echocardiography and cardiac magnetic resonance imaging, and in a patient with right ventricular sarcoma.

Parameter	12.2017 before resection [TTE]	01.2018 after resection before RT [TTE]	01.2018 before RT [CMR]	04.2018 directly after RT [TTE]	06.2019 14 months after RT [TTE]	06.2020 26 months after RT [TTE]
Left atrial area (cm^2^)	18	18	19	24	24	25
Right atrial area (cm^2^)	41	33	30	27	26	25
LV end-systolic diameter (cm)	3.5	2.8	2.9	3.5	3.4	3.4
LV end-diastolic diameter (cm)	5.0	4.7	4.3	4.4	4.8	4.8
Interventricular septum (cm)	1.0	1.0	0.7	1.1	1.2	1.2
LV posterior wall (cm)	0.6	0.8	0.9	1.0	1.2	1.2
LV ejection fraction (%)	63	63	60	55	45	50
LV GLS (%)	nd	nd	nd	-17.4	-16	-15.5
RVOT (cm)	3.8	4.7	nd	3.8	3.7	3.8
RVID (cm)	5.3	5.0	5.5	5.0	4.9	5.0
TAPSE (mm)	9	7	9	12	10	8
S’RV (cm/s)	6	6	7	8	6	4
TR V_max_ (m/s)	4.0	nd	nd	2.2	1.9	1.9

CMR, cardiac magnetic resonance imaging; GLS, global longitudinal strain; LV, left ventricular; nd, no data; RT, radiotherapy; RV, right ventricular; RVID, RV diastolic diameter at the base in the apical 4-chamber view; RVOT, proximal RV outflow tract diameter in the parasternal long-axis view; S’RV, tricuspid annulus systolic velocity in tissue Doppler; TAPSE, tricuspid annular plane systolic excursion; TR, tricuspid regurgitation jet maximum velocity; TTE, transthoracic echocardiography.

**Table 3:** Cardiac dimensions and parameters describing left and right ventricular systolic function in transthoracic echocardiography and cardiac magnetic resonance imaging and in a patient with left atrial sarcoma.

Parameter	9.2016 before resection TTE	11.2016 after resection before ADIC [CMR]	4.2017 after ADIC [CMR]	1.2018 after recurrent resection CMR	6.2018 3 months after RT [CMR]	1.2019 TTE	5.2019 after 3^rd^ cycle of GDXL [TTE]	6.2020 27 months after RT pazopanib TTE
Left atrial area (cm^2^)	25	23	24	24	25	21	22	23
Right atrial area (cm^2^)	nd	26.5	28	25	28	nd	27	20
LV end-systolic diameter (cm)	3.0	2.9	2.8	3.1	3.2	3.4	3.5	4.0
LV end-diastolic diameter (cm)	5.4	5.5	5.3	5.4	5.5	5.4	5.7	5.8
Interventricular septum (cm)	1.1	1.0	1.1	1.1	1.2	1.1	1.2	1.1
LV posterior wall (cm)	1.1	0.9	0.9	0.9	0.9	1.1	1.0	0.9
LV ejection fraction (%)	55	61	54	54	55	57	53	50
RVOT (cm)	3.6	3.2	3.4	3.3	3.4	3.5	4.5	4.0
RVID (cm)	nd	5.1	5.3	5.4	5.1	4.4	4.4	4.5
RV ejection fraction (%)	nd	59	52	52	nd	nd	nd	nd
TR V_max_ (m/s)	nd	nd	nd	nd	nd	3.1	3.2	2.3
RVSP (mmHg)	nd	nd	nd	nd	nd	40	45	24
TAPSE (mm)	nd	nd	nd	nd	Nd	21	17	21
S’RV (cm/s)	nd	nd	nd	nd	nd	nd	12	14

ADIC, chemotherapy with doxorubicin and dacarbazine; CMR, cardiac magnetic resonance imaging; GDXL, chemotherapy with gemcitabin and docetaxel; GLS, global longitudinal strain; LV, left ventricular; nd, no data; RT, radiotherapy; RV, right ventricular; RVID, RV diastolic diameter at the base in the apical 4-chamber view; RVOT, proximal RV outflow tract diameter in the parasternal long-axis view; S’RV, tricuspid annulus systolic velocity in tissue Doppler; TAPSE, tricuspid annular plane systolic excursion; TR, tricuspid regurgitation jet maximum velocity; TTE, transthoracic echocardiography.

#### Patient 2

The first ECHO revealed a 43x28-mm tumor originating in the posterior wall of the LA and infiltrating the posterior part of the mitral annulus. LA and RV were mildly enlarged; otherwise, there were no abnormalities; BNP level was normal and coronary angiography did not show any abnormalities. The patient was discharged with prescribed bisoprolol 2.5 mg QD, amlodipine 5 mg QD, and atorvastatin 40 mg QD, as before surgery. CMR performed 2 months post-surgical excision was clear from local recurrence. After 4 cycles of CTH, with a total doxorubicin dose of 600 mg, ECHO and CMR imaging showed normal LV size and function, and the persistence of RV enlargement, with maintained systolic function (normal RV ejection fraction, TAPSE, and S’RV) (**Table 3**). Ten months after the re-excision of local recurrence (December 2017), sinus rhythm and incomplete RBBB were detected on the ECG. The postoperative courses of RTH and CTH, consisting of five cycles of gemcitabine (800 mg/m^2^) and docetaxel (60-75 mg/m^2^), administered at distant recurrence, were uneventful. Subsequently (May 2019), the patient developed paroxysmal atrial tachycardia (PAT), requiring electrical cardioversion to restore sinus rhythm. A complete RBBB was recorded on the ECG. Pharmacologic therapy was changed; bisoprolol dose was increased to 5 mg QD, and perindopril 5 mg QD was added. During therapy with pazopanib, amiodarone was added due to PAT exacerbation. The patient was free of local sarcoma recurrence and clinically stable, with only the slight worsening of exercise tolerance compared to the pre-disease period. ECHO at 45 months after the diagnosis of sarcoma (27 months after cardiac radiotherapy) showed persistent mild enlargement of the RV (4.5 cm), LA (23 cm^2^), and RA (20 cm^2^), mildly decreased LV systolic function (LVEF 50%), normal LV diastolic function, and preserved RV systolic function. Of note, LV GLS was -16.7%, and peak longitudinal strain was significantly reduced in the basal anterior, lateral, posterior, and (partially) in the mid-ventricular segments (**Figure 1D**). Laboratory tests were within acceptable limits.

## Discussion

In large cohort studies, patients with primary cardiac sarcomas constitute a heterogeneous group that includes a variety of subtypes and primary locations of sarcomas. As primary cardiac sarcomas are rare and treated with a variety of modalities, any retrospective analyses of the impact of either CTH or RTH on outcomes are precluded. In the French Sarcoma Group Study, in which 24 of 124 enrolled patients received radiation, adding RTH was associated with improved progression-free survival (3). Wu et al. reported that among five patients who received postoperative irradiation alone or in combination with chemotherapy, four and one achieved partial and complete remission, respectively (8). There was a trend toward better overall survival in patients receiving any postoperative treatment. In another study, RTH in the dose range of 40–60 Gy was administered to 12 patients with primary cardiac sarcoma. Three of them achieved disease-free survival of 4, 5, and 93 months, respectively (9). However, none of the patients included in these studies was diagnosed with CIS, of which there are only a few case reports involving RTH use (2, 10). While studies on RTH efficacy in CIS are scarce, those on toxicity, especially late effects, remain unavailable.

Herein, we report on two patients with CIS treated with RTH and undergoing regular cardiological control. Based on scarce retrospective reports, the prognosis of patients with primary cardiac sarcoma is dismal, with median overall survival (OS) about 17 months (about 38 months after complete resection, 18 months after incomplete resection, and less than a year in non-resected patients) (3, 4, 8). In our two patients (one with incompletely resected local recurrence and the other with incompletely resected primary tumor), the follow-up period was longer than the expected overall survival—39.5 and 26.5 months since diagnosis and cardiac irradiation, respectively. RTH effectively prevented local recurrence and was well tolerated without acute toxicity symptoms. At the last control visit both patients were in a good general condition, with no early or late RTH-related cardiac complications (e.g. acute pericarditis) despite exceeding the institutional dose constraints for the heart in one of them.

As expected, both patients showed a gradual but slow worsening of LV systolic function (LVEF 50%). STE revealed a significantly reduced LV peak longitudinal strain in the basal and partially mid-ventricular segments, with only mildly reduced global LV strain. The reduction in LV peak longitudinal strain developed within the highest radiation dose (>50 Gy) area. LV diastolic function remained normal. RV enlargement was observed after cardiac surgery, but RV systolic function was normal in the patient with LA sarcoma. In Patient 1, RV systolic function was significantly reduced before surgery, likely due to cancer infiltration and the history of pulmonary embolism. In addition, surgery was more extensive in this patient, and the postsurgical RV defect required the use of a pericardial patch. The regional peak longitudinal strain impairment in the region of the highest RTH dose resembled that seen in Patient 2. However, in this case, the effect of RTH on RV function was unclear. We noted RV hypertrophy in this patient, which could be the result of radiation-related myocardial fibrosis or the sign of compensatory RV remodeling. We did not observe pericardial complications (including pericardial thickening) in either of our patients; however, both patients developed RBBB.

Most previous studies on RIHD involved patients treated with RTH for hematological malignancies or breast cancer; however, no data on the effects of irradiation of the heart sub-volumes have been published to date. It has been shown that radiation causes long-term tissue changes. In the early phase, ionization leads to inflammatory changes with extravasations, edema, and thrombotic state (11–13). This inflammatory phase subsides to a latent fibrotic phase, which is characterized by capillary damage due to endothelial injury, thrombotic lesions and, in the case of the heart, myocardial fibrosis (11–13). The late clinical manifestations of RTH adverse effects include coronary obstruction and premature ischemic heart disease, valvular stenosis and regurgitation, and pericardial and myocardial fibrosis, leading to the constriction and thickening of the LV wall with subsequent diastolic dysfunction as well as conduction abnormalities (14, 15).

Previous studies have shown that the volume of the heart receiving >30 Gy, the mean heart dose of >20 Gy, and the dose per fraction of >2 Gy, is associated with an increased risk of cardiovascular complications (14–17). Meanwhile, it has been shown in breast cancer patients that the risk of major coronary events increases by 7.4% per 1 Gy increase of the mean heart dose, suggesting that the dose-toxicity relationship is continuous without a clearly defined threshold (18). The classic QUANTEC analysis established dose constraints for breast cancer patients with V25 Gy of <10% (dose per fraction = 2 Gy), corresponding to the risk of cardiac mortality of <1%, assessed 15 years post-RTH. The authors also reported the mean pericardium dose of >26 Gy and V30 >46% as risk factors for pericarditis (15). At our institution, we use heart dose constrains of V40 <50% and V60 <25% for chest irradiation other than breast.

LV strain changes after RTH have been previously described; however, their prognostic impact remains unclear (19). Some authors suggest that the decrease in LV GLS persisting 3 years after RTH may indicate permanent LV damage as a result of the fibrotic process (20). The latency period for developing RIHD appears shorter than that reported in previous studies (10–15 years) (21), as 44% of major coronary events attributed to RTH were observed in less than 10 years after irradiation (18).

One of the RIHD manifestations is RV wall thickening, which is considered a late complication (observed at least 5 years after thorax irradiation) (22). Findings on RV systolic function in patients treated with RTH are conflicting (23). It has been shown that the results of TAPSE or S’RV may be falsely underestimated due to geometric rather than structural changes after cardiac surgery (24). Moreover, it has been reported that after RTH, the RV dimension remains unchanged (25, 26). Raina et al. (19) suggested that the larger transverse dimensions of RV (but not RV length) in patients after cardiac surgery may be due to the more spherical shape of RV after surgery. This can explain RV enlargement in one of our patients.

## Conclusion

RTH might be a feasible treatment for cardiac sarcoma, as it is highly effective and relatively safe even at high doses delivered to the heart. However, it remains associated with the risk of RIHD. Due to high-dose radiation delivered directly to the heart, we anticipate that both our patients develop RIHD earlier than suggested by the literature. Therefore, both patients remain under onco-cardiological supervision. Periodic control and transthoracic examinations with modern ECHO techniques (including STE) and CMR continue in both patients.

## Data Availability Statement

The original contributions presented in the study are included in the article/supplementary material. Further inquiries can be directed to the corresponding author.

## Ethics Statement

Ethical review and approval were not required for the retrospective study on human participants in accordance with the local legislation and institutional requirements. The patients/participants provided their written informed consent to participate in this study. Written informed consent was obtained from the individual(s) for the publication of any potentially identifiable images or data included in this article.

## Author Contributions

Research concept and design—AR and RZ. Data collection—AR, HJ, EL, and GS. Data analysis and interpretation—all authors. Writing the first draft of the manuscript—AR. Writing the section on cardiac function—EL and GS. Critical revision of the article—RZ. Final approval of article—all authors. All authors contributed to the article and approved the submitted version.

## Conflict of Interest

The authors declare that the research was conducted in the absence of any commercial or financial relationships that could be construed as a potential conflict of interest.
